# Bibliometric Analysis of Peer-Reviewed Literature on Erosive Tooth Wear From 1945 to 2023

**DOI:** 10.7759/cureus.74830

**Published:** 2024-11-30

**Authors:** Maryam A Alghilan, Ikram UI Haq

**Affiliations:** 1 Department of Restorative and Prosthetic Dental Sciences, King Saud Bin AbdulAziz University for Health Sciences, King Abdullah International Medical Research, Riyadh, SAU; 2 College of Dentistry, King Saud Bin Abdulaziz University for Health Sciences, Riyadh, SAU

**Keywords:** bibliometric review, dental erosion, erosive tooth wear, non-carious cervical lesion, publication trends

## Abstract

Erosive tooth wear (ETW) is a prevalent oral condition with varying etiology, including erosion, abrasion, abfraction, and attrition. It is reported in the literature in different nomenclatures, hindering the ability to identify the emerging trends and influential scholarly works and bodies within this field. Using a bibliometric analysis approach, this study aims to evaluate the trends, themes, and productivity of the research on ETW condition while respecting its different terminologies. The Web of Science database was utilized to obtain the publication records on ETW to implement a retrospective bibliometric study. The data were retrieved on September 10, 2024, with the search terms “Dental erosion” OR “enamel erosion” OR “erosive tooth” OR “dental abrasion” OR “tooth abrasion” OR “toothbrush abrasion” OR “enamel abrasion” OR “non-carious cervical lesion” OR “non carious cervical lesion” OR "abfraction" OR "abfractions" OR “Tooth wear” OR “erosive tooth wear”. We identified 6,069 records, and after applying inclusion/exclusion criteria, we removed 679, and the remaining 5,390 papers were considered for the analysis. The bibliometric indicators include types and accessibility modes, year of publications with citations, publishing sources, most contributing countries, institutions, authorship patterns, top authors, keywords, and the characteristics of the 15 most cited articles were examined. For the data analysis, Microsoft Excel (v.16; Microsoft® Corp., Redmond, WA), VOSviewer (v.1.6.10; https://www.vosviewer.com), and Statistical Product and Service Solutions (SPSS, v.27; IBM SPSS Statistics for Windows, Armonk, NY) software were utilized. The outcome illustrated that 5,390 papers on ETW were contributed by the authors of 127 countries and published in 1,112 journals between 1945 and 2023. The *Journal of Dentistry *published the most papers (n=290), while the *European Journal of Oral Sciences* had the most impactful papers (54.91 citations/paper). Authors from 3,904 institutions participated. The University of São Paulo became the most productive institution with 336 papers, while the University of Bristol’s papers had the maximum citation impact (55.32 citations/paper). About 81.49% of the total authors had produced one paper each. The percentage of average authors for each paper was found to be 4.41, and David Bartlett was the most productive author. The most cited top 15 papers got an average of 297.26 citations per paper. About 77% of research on ETW was published in the last two decades. This bibliometric evaluation provides direction for future research and data regarding the present state of research on ETW.

## Introduction and background

Erosive tooth wear (ETW) is a prevalent condition affecting dental health, characterized by the progressive loss of mineralized tooth surface. It can affect deciduous and permanent teeth with an estimated global prevalence reaching 50% in deciduous teeth and 45% in permanent teeth [1]. The etiology of ETW is multifactorial, including dental erosion (chemical loss of tooth structure by acids), abrasion (mechanical loss of tooth structure by exogenous agent), abfraction (mechanical loss of tooth structure by tooth deflection), and attrition (mechanical loss of tooth structure by tooth-to-tooth friction). ETW is primarily caused by dental erosion, which occurs as a result of teeth exposure to acid of non-bacterial origin [2,3]. The erosive effect of an acidic agent is not only dependent on its acidity characteristics (e.g. pH level) but also the quantity and quality of the protective factors (e.g. dental biofilm) that balance the effects of the erosive acid. The acids involved in ETW can be from extrinsic sources such as dietary acidic foodstuffs and drinks or intrinsic sources such as regurgitation of gastric juices in gastroesophageal reflux disease [4,5]. A growing incidence of ETW has been associated with the modern diet, which often involves increased consumption of acidic drinks and foods [6]. The consequences of ETW may result in increased tooth sensitivity and structural compromises that call for restorative procedures [1,7]. There is a significant development in the field of ETW where research has been progressing considerably since 1945 to date [8,9]. Examination of the body of literature is necessary to track the development of research in ETW, given the growing attention to the topic. The scholarly community must examine the growing body of scientific literature in order to evaluate the impact of research as well as the research findings [10,11].

Bibliometrics is a branch of the quantitative sciences that uses statistical and mathematical techniques to analyze the scientific activity in the field of interest and assess research scholarly works' impact on the scientific community. Furthermore, bibliometrics allows for highlighting the trends and gaps in the research field, which provides an informative insight into the research focus and opportunities, thus helping in directing the efforts toward the areas requiring development.

There are a limited number of bibliometric assessment reports on ETW literature despite the context of a substantial collection of scientific literature about ETW. Marqués Martínez et al. analyzed articles on dental erosion published between 2010 and 2020 and reported the gradual progress of scientific literature in the 20th century, and the exponential growth was recorded in the 21st century [8]. Another bibliometric study examined the attributes of the 100 most cited articles on ETW. These articles were published between the years 1949 and 2015 [9].

The limited recent research profile on ETW, coupled with the different nomenclatures used in the literature for reporting it [12], which hinder the ability to apply a thorough literature analysis, highlight the need for a comprehensive bibliometric analysis on ETW to identify its current state and directs future investigation efforts.

The aim of this bibliometric analysis study is to shed light on various parameters of publications and highlight research trends on ETW, including all possible nomenclature [12] with the long-term goal to identify gaps in current knowledge and provide guidance for future research areas.

## Review

Methods

Study Design

This study was planned as a network analysis using the bibliometric research method and approved by King Abdullah International Medical Research Center, Riyadh, Saudi Arabia. The study examined numerous significant features, including scrutiny of publications, the publication year, frequency of citations, publishing sources, leading countries, institutions, authors, authorship patterns, keywords, and the features of top-15 highly cited articles.

Data Extraction

We executed a computable bibliometric examination using the Web of Science (WoS) database searched on October 6, 2024, with the following search terms: "Dental erosion" OR "enamel erosion" OR "erosive tooth" OR "dental abrasion" OR "tooth abrasion" OR "toothbrush abrasion" OR "enamel abrasion" OR "non-carious cervical lesion" OR “non carious cervical lesion" OR "abfraction" OR “Tooth wear” OR “erosive tooth wear”. The search yields 6,069 records.

Eligibility Criteria

**Figure 1:**
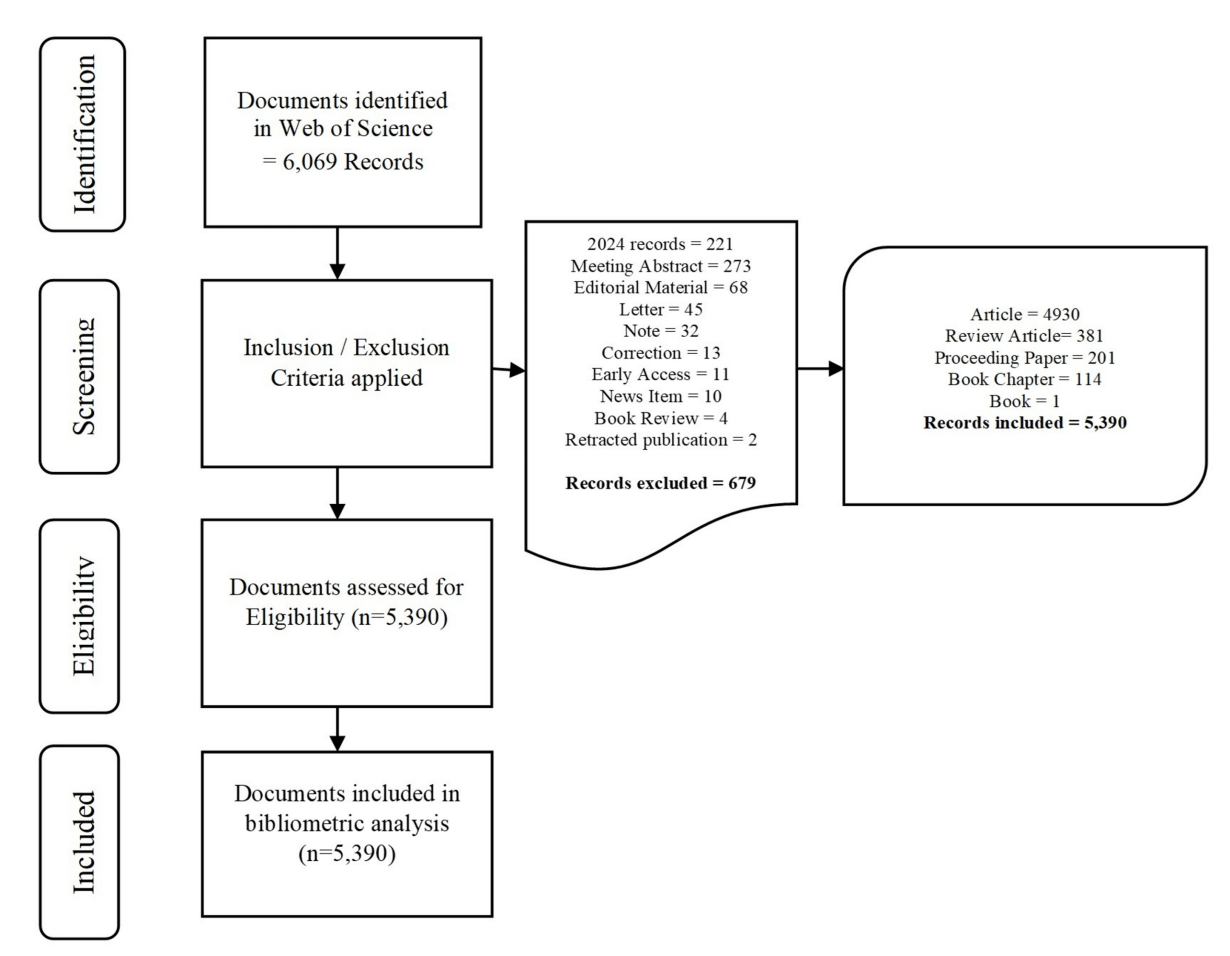
Screening process of the articles

Initially, the study retrieved the dataset of 6,069 documents. Next, we applied the exclusion criteria by removing meeting abstracts, editorial materials, letters, notes, corrections, early access records, news items, book reviews, and records of 2024 as the year is still progressing at the time of data extraction (Figure 1). A total of 679 records were excluded, resulting in 5,390 documents being considered for analysis, which include peer-reviewed literature (Figure 1).

Quality Assessment

The study comprised quality peer-reviewed literature consisting of articles, reviews, proceeding papers, book chapters, and books for analysis and excluded all other documents.

Data Analysis

Microsoft Excel (v.16; Microsoft® Corp., Redmond, WA) software and VOSviewer (v.1.6.10; https://www.vosviewer.com) were utilized for the data analysis [13].

Statistical Analysis

The Statistical Product and Service Solutions (SPSS, v.27; IBM SPSS Statistics for Windows, Armonk, NY) was used to analyze the data of open and subscription-based papers and the papers published in dental and non-dental journals. A p-value of less than 0.05 was applied for statistical significance.

Results

Types of Papers, Accessibility Mode, and Dental and Non-dental Sources

The examination of publication type shows that the majority of papers (n=4,930) were published as original research articles, followed by review articles (n=381), proceeding papers (n=201), book chapters (n=114), and one book.

Regarding mode of accessibility, 37% (n=1,999) of papers were published in open-accessed sources, but these papers gained low citation impact (20.09 cites/paper) as compared to subscription-based papers (n=3,391; 63%), which gained higher citation impact (23.41 cites/paper). The majority of papers (n=2,943; 54.61%) were published in the journals indexed in the WoS category of “Dentistry, Oral Surgery and Medicine”, and these papers got almost double citations (28.12 cites/paper) as compared to papers published in non-dental sources (n=2447; 45.39%) that obtained an average of 20.21 cites/paper (Table 1).

**Table 1:** Accessibility mode and papers in dental/non-dental sources

Variable	Accessibility/Dental and Non-dental Sources	Total Papers	Total Citations	Citation Impact	Statistical Analysis			
					Proportion Difference	Z - Statistic	Standard Error	P-value
Mode of accessibility	Open-Accessed Documents	1999	40163	20.09 cites/doc	0.0332 (0.0284, 0.038)	13.3159	0.0025	<0.001
	Subscription-Based Documents	3931	92062	23.41 cites/doc				
Publications in dental and non-dental journals	Documents Published in Dental Journals	2943	82768	28.12 cites/doc	0.0791 (0.0744, 0.0838)	32.0726	0.0025	<0.001
	Documents Published in Non-Dental Journals	2447	49457	20.21 cites/doc				

The chi-square analysis revealed a highly significant statistical connotation between accessibility modes of articles (open vs. subscription-based access) and number of citations (P=0.001). In addition, the type of journals (dental/non-dental) had a highly statistically significant (P=0.001) effect on papers and citations comparing the dental and non-dental journals. It is established that papers published in subscription-based papers and dental category journals received more citations than open-accessed papers and papers published in non-dental journals.

Frequency of Papers and Citations by Years

The publication period of the selected literature has been spread over 79 years. Only 111 papers were identified in the first 39 years from 1945 to 1983, and moderate progress (n=696) was observed in the next two decades from 1984 to 2003. The second last decade (2004-2013) witnessed promising growth with 1,515 papers. A remarkable publications output was detected in the last decade (2014-2023) with more than half of the papers (n=3,068; 56.92%). About 85% (n=4,583) of the research was published during the last two decades (2004-2023).

**Figure 2:**
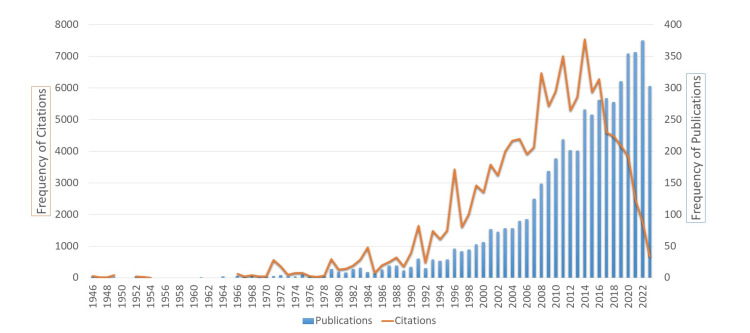
Distribution of papers and citations of ETW research by year ETW: Erosive tooth wear

All these papers were cited 132,225 times with a mean ratio of 22.30 citations per paper. The articles published from 1994 to 2003 obtained the topmost citation impact with 49.37 citations/paper (Figure 2).

Publishing Sources/Journals

The selected papers were published in 1,112 sources of publications, and 663 (59.62%) sources had published a single paper each and 30.09% (n=1622) of the papers have been published in the top 15 journals (Table 2). The *Journal of Dentistry *emerged as the most preferred journal, with 290 papers, followed by the *Caries Research and British Dental Journal *with 208 and 145 papers, respectively. The *Journal of Dentistry *has the highest impact factor (4.8) than *Dental Materials *(4.6). The papers published in the *European Journal of Oral Sciences* got the maximum citation impact (54.91 cites/paper), followed by *Caries Research *(45.21 cites/paper). For recording the impact factor of the journals, we followed the Journal Citation Report (JCR)-2023 available on the WoS database.

**Table 2:** Top 15 publication sources/journals of published research related to ETW

Serial No.	Name of Journal	Impact Factor JCR-2023	Total Articles	Total Citations	Citation Impact
1.	Journal of Dentistry	4.8	290	10179	35.10
2.	Caries Research	2.9	208	9403	45.21
3.	British Dental Journal	2.0	145	4218	29.09
4.	Journal of Oral Rehabilitation	3.1	142	6084	42.85
5.	Archives of Oral Biology	2.2	125	2921	23.37
6.	Clinical Oral Investigations	3.1	117	3489	29.82
7.	Journal of Prosthetic Dentistry	4.3	76	2819	37.09
8.	Australian Dental Journal	1.9	72	2053	28.51
9.	Acta Odontologica Scandinavica	1.4	71	2129	29.99
10.	European Journal of Oral Sciences	1.8	69	3789	54.91
11.	PLOS One	2.9	69	1949	28.25
12.	Dental Materials	4.6	66	2140	32.42
13.	BMC Oral Health	2.6	59	918	15.56
14.	Operative Dentistry	1.4	58	1183	20.40
15.	International Journal of Prosthodontics	2.1	55	1476	26.84

The total link strength analysis based on a number of documents and citations shows that a source named the *Journal of Dentistry *has been found on the top with 8,188 score, followed by the *Caries Research and British Dental Journal *with 7,702 and 3,470 total link strength.

Leading Countries in Erosive Tooth Wear Research

The authors/organizations of 127 countries contributed to ETW research. The details of the top 15 productive countries are shown in Table 3. The authors belonging to the United States produced about one-fifth (n=998; 18.51%) of the total research, followed by England and Brazil with 771 (14.30%) and 764 (14.17%) papers, respectively. Although Canada stood in 15th rank with 124 papers, it got the maximum citation impact (41.28 cites/paper), followed by the Netherlands (39.75 cites/paper).

**Table 3:** Top 15 countries in published erosive tooth wear research

Serial No.	Name of Country	Total Articles	Total Citations	Citation Impact
1.	United States	998	30,676	30.74
2.	England	771	27,092	35.14
3.	Brazil	764	13,475	17.64
4.	Germany	478	16,791	35.13
5.	Switzerland	375	14,191	37.84
6.	China	298	5,751	19.30
7.	Australia	256	7,916	30.92
8.	Italy	225	5,920	26.31
9.	Netherlands	198	7,871	39.75
10.	Japan	192	4,364	22.73
11.	Spain	179	3,812	21.30
12.	India	165	1,461	8.85
13.	France	156	3,809	24.42
14.	Norway	136	4,329	31.83
15.	Canada	124	5,119	41.28

Leading Research Organizations 

The co-author’s analysis of affiliated organizations stated that 3,904 organizations were involved in ETW research. Collaborative publications among organizations in ETW research were reported by 429 organizations. The University of São Paulo had the highest total link strength with 303, followed by the University of Zurich and the University of Bern with 248 and 223 link strength, respectively.

Table 4 presents the details of the top 15 productive organizations. The University of São Paulo stands out with 336 articles, followed by the University of London and King’s College London with 284 and 197 papers, respectively. The research produced by the authors of the University of Bristol gained the highest citation impact (46.65 cites/paper), followed by Vrije Universiteit Amsterdam (49.68 cites/paper), and Academic Center of Dentistry Amsterdam (49.55 cites/paper). Of the top 15 organizations, four belonged to England; three from the Netherlands; two each from Brazil, Switzerland, and the United States; and one each from France and Norway.

**Table 4:** Top 15 organizations in ETW research

Serial No.	Affiliation	Total Articles	Total Citations	Citation Impact
1.	University of São Paulo, Brazil	336	6587	19.60
2.	University of London, England	284	9439	33.24
3.	King’s College London, England	197	6494	32.96
4.	University of Zurich, Switzerland	164	5782	35.26
5.	University of Bern, Switzerland	163	7604	46.65
6.	Universidade Estadual Paulista, Brazil	138	2367	17.15
7.	University of Bristol, England	115	6368	55.32
8.	Indiana University, United States	113	3023	26.75
9.	Centre National De La Recherche Scientifique CNRS, France	94	2184	23.23
10.	Ranboud University Nijmegen, Netherlands	89	3258	36.61
11.	University of California System, United States	87	2987	34.33
12.	Vrije Universiteit Amsterdam, Netherlands	85	4223	49.68
13.	Academic Center for Dentistry Amsterdam, Netherlands	84	4162	49.55
14.	University of Oslo, Norway	82	2952	36.00
15.	Guy’s St. Thomas NHS Foundation Trust, England	73	2801	38.37

Authorship Patterns 

**Figure 3:**
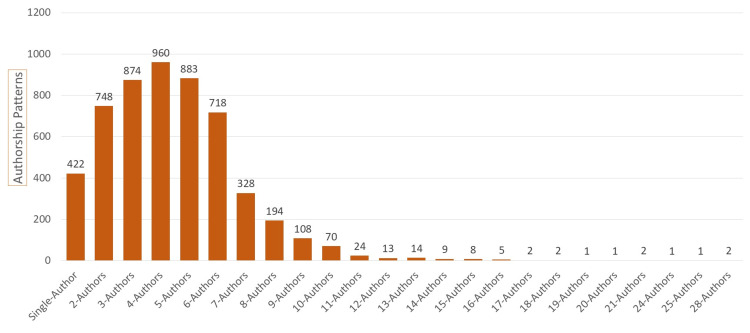
Authorship patterns in ETW research publications ETW: Erosive tooth wear

The analysis of authorship patterns reveals that four-author collaboration was found most common (n=960; 17.81%), followed by five-author and three-author collaboration with 883 and 874 papers, respectively. About two-thirds (n=3,435; 63.79%) of the papers were written by three- to six-author groups. The ratio of the average number of authors per paper increased twofold, rising from 2.63 authors per paper between 1945 and 1999 to 4.41 authors per paper in subsequent years. Between 2000 and 2010, this proportion reached 3.76 authors per paper then increased to 4.87 authors per paper from 2011 to 2023. This proportion increased to 3.76 authors per paper from 2000 to 2010 and reached 4.87 authors per paper from 2011 to 2023 (Figure 3).

*Top Productive Authors* 

The details of the top 15 authors are shown in Table 5. David Bartlett of King’s College London emerged as the most prolific author with 123 papers, and Adrain Lussi of the University of Bern secured the second rank with 112 papers. Carolina Ganss of Philipps University of Marburg was found as most impactful author based on citation impact (67.02 cites/paper), followed by Nicola X. West of the University of Bristol (59.64 cites/paper). The top 15 authors belonged to six countries, five authors belonged to Brazil, and interestingly all are affiliated to the University of São Paulo. Afterward, four authors from Switzerland, two each from England and Germany, and one author each belonged to the United States and the Netherlands.

**Table 5:** Top 15 authors in ETW research ETW: Erosive tooth wear

Serial No.	Name	Affiliation	Total Articles	Total Citations	Citation Impact
1.	David Bartlett OR David W. Bartlett	King’s College London, England	123	5041	40.98
2.	Adrian Lussi	University of Bern, Switzerland	112	5647	50.42
3.	Marilia Afonso Rabelo Buzalaf	University of São Paulo, Brazil	90	2930	32.56
4.	Thomas Attin	University of Zurich, Switzerland	86	3744	43.53
5.	Anderson T Hara	Indiana University, United States	70	1604	22.91
6.	Ana Carolina Magalhaes	University of São Paulo, Brazil	63	2168	34.41
7.	Annette Wiegand	University of Gottingen, Germany	60	2645	44.08
8.	Thago Saads Carvalho	University of Bern, Switzerland	59	1696	28.75
9	Daniela Rios	University of São Paulo, Brazil	58	1526	26.31
10.	Marie-Charlotte Huysmans	Radboud University Nijmegen Medical Center, Netherlands	54	2142	39.67
11.	Carolina Ganss	Philipps University of Marburg, Germany	52	3485	67.02
12.	Tais Scaramucci	University of São Paulo, Brazil	52	755	14.52
13.	Marcus Clauss	University of Zurich, Switzerland	49	1296	26.45
14.	Nicola X. West	University of Bristol, England	47	2803	59.64
15.	Heitor Marques Honorio	University of São Paulo, Brazil	45	782	17.38

Co-occurrence of Author’s Keyword 

**Figure 4:**
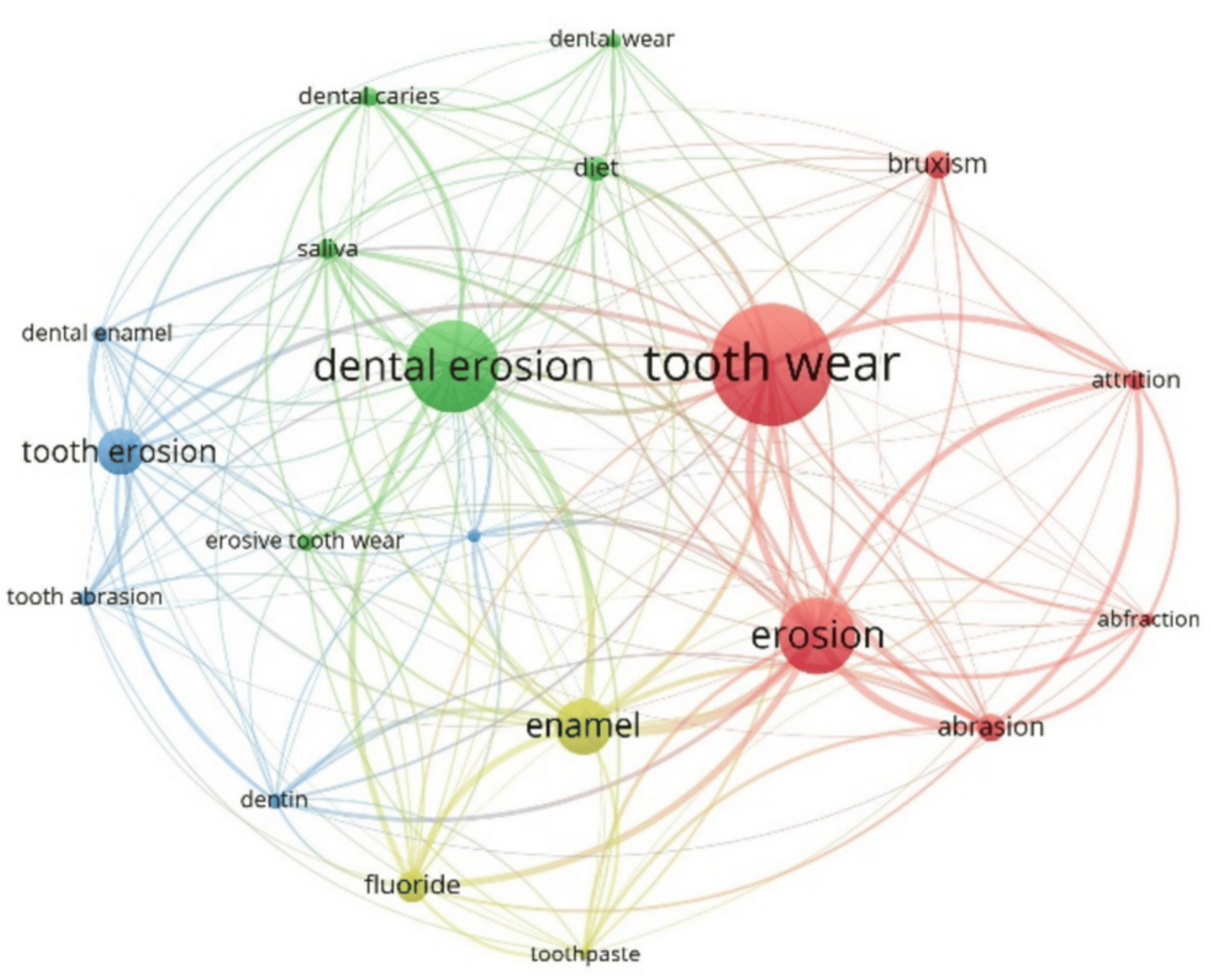
Co-occurrence network analysis of the top 20 keywords in ETW research publications ETW: Erosive tooth wear

Table 6 and Figure 4 present the details and network analysis of the top 20 author’s keywords. These keywords have been distributed in four clusters by VOSviewer software; the first cluster has six keywords (abfraction, abrasion, attrition, erosion, bruxism, and tooth wear), there are six keywords in the second cluster (dental caries, dental erosion, dear wear, diet, erosion dental wear, and saliva), and there are five keywords in the third cluster (demineralization, dentin enamel, dentin, tooth abrasion, tooth erosion). Three keywords, enamel, fluoride, and toothpaste come in the fourth cluster. The keyword of “tooth wear” occurred most frequently, followed by “dental erosion” and “erosion”. Meanwhile, “erosion” has the maximum total link strength, followed by “enamel” and “tooth wear”.

**Table 6:** Top 20 frequently occurred keywords in ETW publications ETW: Erosive tooth wear

Serial No.	Keyword	Occurrences	Total Link Strength
1.	Tooth wear	747	492
2.	Dental erosion	555	340
3.	Erosion	458	613
4.	Enamel	337	497
5.	Tooth erosion	273	233
6.	Fluoride	181	272
7.	Abrasion	161	316
8.	Bruxism	158	86
9.	Diet	141	101
10.	Saliva	121	173
11.	Attrition	108	200
12.	Dental caries	108	99
13.	Erosive tooth wear	95	84
14.	Tooth abrasion	92	82
15.	Dental enamel	91	108
16.	Dentin	87	151
17.	Dental wear	86	51
18.	Demineralization	73	113
19.	Abfraction	69	106
20.	Toothpaste	69	119

Top-Cited Papers 

The top 15 papers were cited 4,459 times, with an average of 297.26 citations per paper. The analysis of the paper’s types stated that six papers consisted of original research articles, five proceedings papers, followed by three review articles, and one book chapter (Appendix, Table 7). These top-cited articles were published between the years 1991-2018. Apart from book chapters, 14 papers were published in 10 journals, and three were papers published in the *European Journal of Oral Sciences*, followed by two papers in *Caries Research *and *Clinical Oral Investigations*.

Discussion

We studied the progression of research published in ETW over a span of 79 years (1945-2023) applying a comprehensive quantitative technique for bibliometrics.

The WoS database used in this study is an excellent option for performing bibliometric evaluation due to its broad coverage and strong features. WoS has a wide range of journals from several fields, such as the medical and dental sciences. Its extensive citation data is one of its best qualities. This enables scholars to monitor the frequency of citations received by a certain piece of work, offering valuable perspectives on its impact and influence within the scholarly community. WoS makes interdisciplinary study easier by indexing publications from a variety of disciplines [14,15]. This is particularly significant for subjects such as ETW since they can have connections to gastroenterology, public health, and nutrition [9]. Acquiring a thorough, dependable, and sophisticated grasp of research trends and impacts requires bibliometric analysis using the WoS database [16].

The study exhibits remarkable findings, such as papers published in subscription-based (closed-assessed) sources that gained a higher ratio of citations (23.41 cites/paper). Similarly, the papers published in journals related to the dental category achieved more citations (28.12 cites/paper). On the other hand, the ETW research published in open-accessed format and non-dental journals received less attention from the scholarly community. The papers published from 1994 to 2003 gained the maximum citation impact (49.37 cites/paper), and it is likely to undertake that papers published during the recent 10 years had the least impact from citations because the citation rates often increase gradually [17].

The current study is imperative because it provides valuable information for evaluating the publication growth of ETW. It supplements our understanding of the present state of ETW in preventive and restorative dentistry. More than 1,100 publication channels have been identified and 30% of the papers were published in the top 15 sources. The *Journal of Dentistry *appears to be the most targeted journal as it has the highest number of papers, followed by *Caries Research*. In another view, the *European Journal of Oral Sciences* published the most impactful papers, followed by *Caries Research*. In line with this finding, Rocha et al.'s study on the analysis of the 100 most cited papers on ETW revealed that *Caries Research* was the most preferred journal, followed by the *Journal of Dentistry* [9]. This finding shows that the authors have been targeting the readership of these journals.

Our data show that the authors belonging to 127 countries contribute to ETW research. Despite the international scope, the majority of research has come from developed countries, and one-fifth (18.51%) of the research produced by the United States, England, and Brazil, followed narrowly behind, with 14.30% and 14.17% papers, respectively. Corresponding with this research, Marqués Martínez et al.'s study analyzed PubMed-indexed 1,090 articles on dental erosion, and most of the papers were contributed by Brazil, followed by England and the United States [8]. In our study, the United States contributed the most papers, the Netherlands achieved the rank of highest citation impact (39.75 cites/paper), and Switzerland came in just after with 37.84 cites/paper. Brazil secured reasonable status, and the University of São Paulo of Brazil was found to be the most active research-producing organization. The University of London and King’s College London secured the second and third ranks, respectively. Out of 15 top organizations, four belonged to England. However, Vrije Universiteit Amsterdam had the highest impact, as demonstrated by the number of citations per paper. Another bibliometric study on *Dental Caries *also stated that the University of São Paulo had a most prolific organization [18]. Most of the research on ETW has been generated from developed nations because of the quality of their institutions and research centers, as well as an adequate allocation of funds for research [19].

In the analysis of authorship patterns, our study emphasizes the prevalence of research collaboration, and only a small segment of papers (7.82%) were authored by a single researcher. About two-thirds of the papers (64%) were contributed by three- to six-author collaboration. Another study reported that the mean ratio of authors per article was 4.7 ± 2.1, and this ratio increased from 4.2 in 2011 to 5.6 in 2020 [8]. Our study also supports the fact that the average number of authors per paper increased from 2.63 authors from 1945 to 1999, to 4.87 authors from 2011 to 2023. Remarkably, David Bartlett from King’s College London, followed by Adrian Lussi from the University of Bern, authored more than 100 papers each, while Caroline Ganss emerged as the most dominant based on citation count. Another bibliometric study on dental erosion to support this finding is that Adrian Lussi was found most productive, and his articles were cited with a mean ratio of 117.28 cites/article [8].

The study performed a co-occurrence analysis of keywords that indicate that “tooth wear” was found to have the most occurred keyword with an occurrence rate of 747, followed by “dental erosion” and “erosion”. Keyword analysis helps identify the thematic landscape of the scientific literature. Rocha et al. performed the keywords co-occurrence analysis of 100 most cited papers on ETW, and the study had almost similar findings where “dental erosion” was found most occurred keyword, followed by “erosion” and “enamel” [9]. Marqués Martínez et al. performed the subject dispersion on dental erosion research. It showed that “preventive treatment” was a dominant area of research, followed by “diet” and “hard tissues”. “Socioeconomic level” and “soft tissues” were discovered in the less-researched areas [8].

The analysis of citations discloses a concentration of impact within 15 highly influential papers, with an impressive average of 297.26 citations per paper. These papers were published in 28 years from 1991 to 2018, seven of them appeared before 2000, and eight were published after 2000. In addition, the top 15 most cited papers originated from a varied cluster of authors, across 15 countries. However, Switzerland was found at the top of the list with the most six papers, followed by the United States with four papers. It is significant to note that these top cited papers were published across many different sources, with the *European Journal of Oral Sciences* taking the lead with three papers, followed by *Caries Research* and *Clinical Oral Investigations* with two papers each. Rocha et al. examined the bibliometric attributes of the 100 most cited articles on ETW, and these papers were cited with an average of 97.44 cites/article and published between the years 1949 and 2015. These articles were contributed by 13 countries, and 34 articles were contributed by England, followed by Germany and Switzerland with 18 and 10 articles, respectively [9].

One of the limitations of this study is the dependency on particular keywords for identifying scientific literature may have led to the omission of pertinent research that did not employ such terms. Further studies using a broader keyword could reveal a deeper knowledge of the ETW research output. Moreover, we included selected types of documents from the dataset, retrieved from WoS. However, future researchers may include all indexed records and records in other databases such as PubMed and Scopus, and the inclusion of gray literature will be more beneficial for a broader and deeper understanding of the subject. Additionally, the study was mostly quantitative and focused more on numerical data than on assessing the methodological stature of particular studies. Thus, it will be advantageous to conduct additional research evaluating the quality in light of the level of evidence about ETW.

## Conclusions

About 77% of research on ETW was published in the last two decades. More than 1,100 publication channels have been identified, and 30% of the papers were published in the top 15 sources. The bibliometric evaluation of ETW exposed a noteworthy growth of research; identified the prominent contribution to the field at the level of authors, institutions, and countries; and highlighted a significant impact of research collaboration and the targeted efforts on studying different aspects of ETW.

## Appendices

**Table 7:** Fifteen most cited publications in ETW ETW: Erosive tooth wear

Serial No.	Bibliographic Detail of Paper	Total Citations	Citation Density by Year (Rank)
1.	International consensus on the assessment of bruxism: Report of a work in progress [20]	669	95.57 [1]
2.	Epidemiology of bruxism in adults: a systematic review of the literature [21]	376	31.33 [2]
3.	Risk factors in dental erosion [22]	360	10.59 [10]
4.	Basic Erosive Wear Examination (BEWE): a new scoring system for scientific and clinical needs [23]	347	20.41 [5]
5.	Dental erosion--an overview with emphasis on chemical and histopathological aspects [24]	321	22.93 [4]
6.	Dental erosion. Definition, classification and links [5]	310	10.69 [9]
7.	Etiology of dental erosion--extrinsic factors [25]	291	10.03 [11]
8.	Erosive tooth wear: a multifactorial condition of growing concern and increasing knowledge [6]	264	24.00 [3]
9.	Attrition, abrasion, corrosion and abfraction revisited: a new perspective on tooth surface lesions [26]	225	10.71 [8]
10.	Dental erosion in a population of Swiss adults [27]	223	6.56 [14]
11.	Non-carious cervical lesions [28]	220	6.47 [15]
12.	Saliva and dental erosion [29]	216	16.62 [6]
13.	Erosion--diagnosis and risk factors [30]	215	12.65 [7]
14.	Enamel erosion by some soft drinks and orange juices relative to their pH, buffering effect and contents of calcium phosphate [31]	211	8.12 [12]
15.	Pathogenesis and modifying factors of dental erosion [32]	211	7.28 [13]
